# Clinical evaluation of rare copy number variations identified by chromosomal microarray in a Hungarian neurodevelopmental disorder patient cohort

**DOI:** 10.1186/s13039-022-00623-z

**Published:** 2022-11-01

**Authors:** Anna Lengyel, Éva Pinti, Henriett Pikó, Árvai Kristóf, Tünde Abonyi, Zaránd Némethi, György Fekete, Irén Haltrich

**Affiliations:** 1 II. Department of Pediatrics, Semmelweis University, Budapest, Hungary; 2 Department of Internal Medicine and Oncology, Semmelweis University, Budapest, Hungary

**Keywords:** Neurodevelopmental disorders, Chromosomal microarray, 14q11.2 microdeletion, Variants of unknown significance, PPP3CA, SYNDIG1

## Abstract

**Background:**

Neurodevelopmental disorders are genetically heterogeneous pediatric conditions. The first tier diagnostic method for uncovering copy number variations (CNVs), one of the most common genetic etiologies in affected individuals, is chromosomal microarray (CMA). However, this methodology is not yet a routine molecular cytogenetic test in many parts of the world, including Hungary. Here we report clinical and genetic data of the first, relatively large Hungarian cohort of patients whose genetic testing included CMA.

**Methods:**

Clinical data were retrospectively collected for 78 children who were analyzed using various CMA platforms. Phenotypes of patients with disease-causing variants were compared to patients with negative results using the chi squared/Fisher exact tests.

**Results:**

A total of 30 pathogenic CNVs were identified in 29 patients (37.2%). Postnatal growth delay (p = 0.05564), pectus excavatum (p = 0.07484), brain imaging abnormalities (p = 0.07848), global developmental delay (p = 0.08070) and macrocephaly (p = 0.08919) were more likely to be associated with disease-causing CNVs.

**Conclusion:**

Our results allow phenotypic expansion of 14q11.2 microdeletions encompassing *SUPT16H* and *CHD8* genes. Variants of unknown significance (n = 24) were found in 17 patients. We provide detailed phenotypic and genetic data of these individuals to facilitate future classification efforts, and spotlight two patients with potentially pathogenic alterations. Our results contribute to unraveling the diagnostic value of rare CNVs.

**Supplementary Information:**

The online version contains supplementary material available at 10.1186/s13039-022-00623-z.

## Background

Neurodevelopmental disorders (NDDs), including intellectual disability (ID), global developmental delay (GDD), autism spectrum disorder (ASD), attention deficit/hyperactivity disorder (ADHD), specific learning disorders, motor disorders (developmental coordination disorder, stereotypic movement disorder, etc.), and communication disorders (language disorder, speech sound disorder, etc.) pose a considerable challenge in everyday pediatric practice. Children with these conditions face difficulties in adaptive functions, social interactions and self-care, which may translate to entire families under serious emotional (and often financial) stress. Early and accurate diagnosis is therefore not only important for optimal care of the affected child, but for alleviating the burden and guilt on the parents.

The etiology underlying NDDs is exceedingly complex, and genetic underpinnings show further heterogeneity. One of the largest groups of genetic etiologic factors are copy number variations [CNVs, a few dozen kilobase pair (Kb) to several megabase pair (Mb) large structural variations in the genome]. The recommended gold standard techniques for diagnosing such alterations (excluding those that cause clinically recognizable syndromes) are chromosomal microarray analyses (CMA) - array comparative genomic hybridization (array CGH) and single nucleotide polymorphism (SNP) arrays. These methodologies have elevated the diagnostic yield of traditional cytogenetics from approximately 5% to 15–20% [1–4]. CMA is however not yet a part of routine genetic testing in Hungary. Delineating clinical features that could help with indicating this laborious test would be beneficial to both families and clinicians. Here we report our experiences with the first relatively large Hungarian pediatric cohort who underwent CMA testing.

## Results

During the period between 2011 and 2020, 88 patients of Semmelweis University’s II. Department of Pediatrics underwent CMA testing. Children were selected for investigation if they had idiopathic DD/ID or a major congenital anomaly, and at least one additional suggestive feature (other NDDs, characteristic facies, multiple congenital anomalies, etc.), and the family consented to further genetic testing. At our Department, the typical DiGeorge syndrome (proximal A-D 22q11.11 deletion, including *TBX1* gene), Williams syndrome and the deletion form of Prader-Willi syndrome are diagnosed by fluorescent *in situ* hybridization (FISH), therefore these cases are not included in this study. 78 patients underwent CMA after negative routine cytogenetic evaluations, while 10 patients were tested to refine the findings of previous tests. These latter patients with microscopically visible rearrangements (genetic and phenotypic data listed in Additional File 1) have been excluded from further statistical analysis.

The final investigation cohort consisted of 78 individuals, of which 47 were males (60.2%; male:female ratio 1.52:1). The average age at first clinical genetics consultation was 4.17 years (median 2.00 years, range from 4 days old to 20 years 7 months old). Disease-causing variants (n = 30) were identified in 29 patients (15 males and 14 females), which translates to an overall diagnostic yield of 37.18%. The disease-causing CNVs were on average 3.481 Mb large (median 1.124 Mb); 19/30 were deletions and 11/30 were duplications (Additional file 2). Chromosome 16 was most frequently affected (9/30; 30.0%), followed by chromosome 22 (4/30; 13.3%), and chromosomes 14, 17 and X (3/30; 10.0% each) (Additional file 3).

Information regarding the disease-causing CNVs is detailed in Table 1, the accompanying phenotypes of the patients are elaborated in Table 2. One child (patient SEG2_82) carried multiple disease-causing CNVs, specifically a terminal duplication of chromosome 19 (57243585_58445449; GRCh38) and a terminal deletion of chromosome 22 (49368551_50759338; GRCh38). These rearrangements originated from a paternal balanced translocation, and subsequent targeted FISH analyses revealed the CNVs in the patient’s younger sister as well. Of further note are patients SEG2_62 and SEG2_68. The former carried a deletion of chromosome X (6537108_8167604 Mb; GRCh37) encompassing the *STS* gene (OMIM*300,747), and was subsequently diagnosed with a mild form of X-linked ichthyosis (OMIM#308,100). An additional finding on the SNP array was a large loss of heterozygosity (LOH) on chromosome 14 (20052038_106871264 Mb; GRCh37). This finding was followed up by methylation analysis and maternal uniparental disomy 14 (Temple syndrome, OMIM#616,222) was confirmed, which was ultimately the underlying cause of his primary phenotype (see Table 2). SEG2_68’s parents are first cousins, which resulted in the identification of LOH on 21 regions of 12 chromosomes (overall 196.8 Mb of the genome).

**Table 1:** **Disease-causing CNVs identified in the presented patient group listed by chromosome**

Copy Number Variation	GRCh	Patient	Size (Kb)	Class.	Dosage Sensitivity^#^	Candidate genes (number of protein coding genes within CNV)	Syndrome/disorder
1p36.33p36.22(820001_9348000)x1	37	SEG2_52	8528.0	P	HI: 3; TS: 2	*KLH17, HES4, AGRN, VWA1, MMP23B, CDC2L2, GABRD, SKI, PLCH2, CAMTA1 (HI: 3, TS: 0)* (111)	1p36 microdeletion [OMIM#607,872, (44)]
2q23.1(149060704_149313819)x3	37	SEG2_44	253.1	LP	-	*MBD5 (HI: 3, TS: 1)* (1)	2q23.1 microduplication (49)
4q32.2q35.2(161869551_190790881)x1	37	SEG2_70	28921.3	P	-	*CLCN3, HAND2, TEN3* (84)	4q32.2q35.2 deletion (51)
8p23.1(7117851_11969155)x1	37	SEG2_50	4851.3	P	HI: 3, TS: 2	*GATA4* (48)	8p23.1 microdeletion (ORPHA:251,071)
14q11.2(21414942_21966929)x1	37	SEG2_49	552.0	LP	HI: 2, TS: 0	*CHD8, SUPT16H* (19)	14q11.2 microdeletion (20)
14q11.2(21438704_22101647)x1	37	SEG2_68	662.9	LP	HI: 2, TS: 0	*CHD8, SUPT16H* (21)	14q11.2 microdeletion (20)
14q11.2(21511829_22131455)x1	37	SEG2_89	619.6	LP	HI: 2, TS: 0	*CHD8, SUPT16H* (18)	14q11.2 microdeletion (20)
15q11.2q13.1(22765628_29060493)x1	37	SEG2_33	6294.9	P	HI: 3; TS: 3	*UBE3A, SNRPN* (22)	Angelman syndrome (OMIM#105,830)
16p13.3(3263725_4309863)x1	38	SEG2_5	1046.1	P	HI: 3; TS: 1	*CREBBP* (20)	16p13.3 microdeletion (OMIM#610,543)
16p12.2(21953152_22480514)x3	37	SEG2_32	527.4	LP	HI: 2, TS: 0	*UQCRC2, EEF2K, CDR2* (7)	16p12.2 microduplication (39)
16p11.2(28824802_29040571)x1	37	SEG2_53	215.8	P	HI: 3; TS: 1	*SH2B1* (9)	Distal 16p11.2 microdeletion (OMIM#613,444)
16p11.2(29620689_30190568)x3	37	SEG2_17	569.9	P	HI: 3; TS: 3	*KIF22, MAZ, PRRT2, KCTD13, TBX6, MAPK3* (26)	16p11.2 microduplication (OMIM#614,671)
16p11.2(29624765_30199351)x3	37	SEG2_26	574.6	P	HI: 3; TS: 3	*KIF22, MAZ, PRRT2, KCTD13, TBX6, MAPK3* (27)	16p11.2 microduplication (OMIM#614,671)
16p11.2(29624765_30199351)x3	37	SEG2_27	574.6	P	HI: 3; TS: 3	*KIF22, MAZ, PRRT2, KCTD13, TBX6, MAPK3* (27)	16p11.2 microduplication (OMIM#614,671)
16p11.2(29656684_30190568)x1	37	SEG2_39	533.9	P	HI: 3; TS: 3	*KIF22, MAZ, PRRT2, KCTD13, TBX6, MAPK3* (26)	Proximal 16p11.2 microdeletion (OMIM#611,913)
16q12.2q21(56340118_60294492)x1	37	SEG2_85	3954.4	LP	-	*GNAO1 (HS:1, TS: 0)* (55)	16q12.2q21 microdeletion (40)
16q22.2q23.3(72155844_82148404)x1	37	SEG2_37	9992.6	LP	-	*WWOX, MAF, CMIP, CNTNAP4* (49)	16q22.2q23.3 microdeletion [OMIM#614,541, (41)]
17p13.3(7_2084490)x1	37	SEG2_30	2084.5	LP	-	*CRK, YWHAE* (34)	17p13.3 microdeletion syndrome (42,43)
17p11.2(16727490_20433502)x1	37	SEG2_59	3706.0	P	HI: 3; TS: 3	*RAI1, FLCN* (49)	Smith-Magenis syndrome (OMIM#182,290)
17q12(34835983_36243365)x3	37	SEG2_87	1407.4	P	HI: 3; TS: 3	*HNF1B* (15)	17q12 microduplication (OMIM#614,526)
18q22.1q23(69071896_80256240)x1	38	SEG2_21	11184.3	P	-	*RTTN, SOCS6, CYB5A, ZNF407, TSHZ1* (33)	18q22.1q23 microdeletion (OMIM#601,808)
19p13.3(753219_1477508)x3	37	SEG2_15	724.3	LP	-	*STK11, APC2*	19p13.3 microduplication (ORPHA:447,980)
19q13.43(57243585_58445449)x3	38	SEG2_82*	1201.9	P	-	*RPS5* (50)	19q13.43 microduplication (45,46)
22q11.21q11.22(21460640_22962962)x1	37	SEG2_80	1502.3	P	HI: 3; TS: 3	*MAPK1* (19)	22q11.21q11.22 microdeletion (47)
22q11.21q11.22(21934268_22336871)x3	37	SEG2_7	402.6	P	HI: 3; TS: 3	*MAPK1* (9)	22q11.2 microduplication [OMIM#608,363, (48)]
22q13.33(49368551_50759338)x1	38	SEG2_82*	1390.8	P	-	*SHANK3 (HI: 3, TS: 0)* (34)	Phelan-McDermid syndrome (OMIM#606,232)
22q13.33(50971316_51224252)x3	37	SEG2_12	252.9	LP	-	*SHANK3 (HI: 3, TS: 0)* (9)	22q13 microduplication (OMIM#615,538)
Xp22.33(566719_807207)x3	37	SEG2_61	240.5	P	-	*SHOX* (1)	Léri-Weill dyschondrosteosis (OMIM#127,300), idiopathic short stature (OMIM#300,582)
Xp22.31(6537108_8167604)x0	37	SEG2_62	1630.5 *86819.2*	P	HI: 3; TS: 40	*STS* (5)	X-linked ichtyosis (OMIM#308,100)
Xq22.1q23(101597527_111626047)x1	38	SEG2_78	10028.5	P	-	*PLP1 (HI: 3, TS: 3)* (79)	Xq22 microdeletion (50)

*: Patient with more than one disease-causing copy number variation (CNV); Kb: kilobase; Class.: classification; P: pathogenic; LP: likely pathogenic; OMIM: Online Mendelian Inheritance in Man;

^#^Based on ClinGen Dosage Sensitivity Curations (https://clinicalgenome.org). HI: haploinsufficiency; TS: triplosensitivity;

3: Sufficient Evidence

2: Emerging Evidence

1: Little Evidence

0: No evidence

40: Dosage Sensitivity Unlikely

**Table 2:** **Phenotypes of patients with disease-causing CNVs**

Patient	Sex	Syndrome/disorder	Detailed phenotype
SEG2_5	F	16p13.3 microdeletion (OMIM#610,543)	Microcephaly, small forehead, retrognathia, hypotelorism, narrow and depressed nasal bridge, low-set ears, abnormality of the pinna, high palate, enlarged thorax, pectus excavatum, pollex duplex, broad thumbs with radial deviation, broad 1st toes, low anterior hairline, decreased fetal movement, congenital glaucoma, atrial SD, moderate left ventricular hypertrophy, PS, laryngospasm
SEG2_7	M	22q11.2 microduplication [OMIM#608,363, (48)]	Premature birth, complicated perinatal adaptation, neonatal hypoglycaemia, bark-like cry, maternal alcohol consumption during pregnancy possible, somatic DD, ID/DD, microcephaly, small forehead, unilateral narrow palpebral fissure and microphthalmia, hypoplastic philtrum, high palate, unilateral clinodactyly of the 5th finger, unilateral STPC, pectus excavatum, rectus diastasis, dystrophic toenails, MuHy, brain MRI: CCH, hypoplastic hippocampus, PDA and atrial SD, inguinal hernia, hypospadias
SEG2_12	M	22q13 microduplication (OMIM#615,538)	Global DD/ID, severe speech DD, relative macrocepahly, broad face and forehead, hypertelorism, high palate, widely spaced teeth, JH, cutis laxa, imbalance, autistic features
SEG2_15	M	19p13.3 microduplication (ORPHA:447,980)	Somatic DD, feeding difficulties, global DD/ID, microcephaly, micro- and retrognathia, low-set, prominent and simple ears, hypoplastic philtrum, narrow mouth, rib anomaly, pectus excavatum, JH, coarse hair, low anterior hairline, brain MRI: mild cerebral atrophy, generalized MuHy, unilateral coloboma, stenosis of the nasolacrimal duct, bilateral sensorineural hearing impairment (familial), duodenal atresia, cholestasis, splenomegaly, impaired liver function, bicuspid aortic valve, cryptorchidism, hypospadias, hypocortisolism
SEG2_17	M	16p11.2 microduplication (OMIM#614,671)	Global DD, severe speech DD, developmental regression, facial asymmetry, prominent forehead, epicanthus, wide and depressed nasal bridge, low-set and prominent ears, high palate, clinodactyly of 3rd -5th toes, brain CT: cerebral atrophy, ventriculomegaly, generalized MuHy, autistic features, poor attention, temper tantrums, abnormal eating behavior, strabismus, cryptorchidism
SEG2_21	F	18q22.1q23 microdeletion (OMIM#601,808)	SGA, feeding difficulties, global DD, relative macrocepahly, broad forehead, micrognathia, epicanthus, depressed nasal bridge, overlapping toes, high anterior hairline, brain MRI: ventriculomegaly, delayed myelinization, nystagmus, MuHy, lower limb muscular hypotrophy, reduced tendon reflexes, atrial SD
SEG2_26	M	16p11.2 microduplication (OMIM#614,671)	Global DD/ID, prominent forehead, micrognathia, down-slanted palebral fissures, hypertelorism, absent medial eyebrows, low-set and prominent ears, downturned corners of the mouth, epilepsy, ASD, poor attention, strabismus, caries
SEG2_27	M	16p11.2 microduplication (OMIM#614,671)	Global DD/ID, prominent forehead, down-slanted palebral fissures, hypertelorism, absent medial eyebrows, low-set and prominent ears, downturned corners of the mouth, JH, generalized MuHy, ASD
SEG2_30	F	17p13.3 microdeletion syndrome (42,43)	Prematurity, somatic DD, global DD/ID, large fontanels, delayed closure of fontanels, triangular face, high, broad and prominent forehead, microphthalmia, wide and depressed nasal bridge, low-set ears, thin upper lip vermillion, downturned corners of the mouth, short philtrum, high palate, pectus excavatum, small hands, brachydactyly, TPC, low posterior hairline, brain MRI: CCH, ventriculomegaly, aqueduct stenosis, arachnoid cysts, hydrocephalus, tethered cord, strabismus, delayed eruption of teeth, delayed bone age, atrial SD, anteriorly placed anus
SEG2_32	F	16p12.2 microduplication (39)	Global DD/ID, speech regression, epicanthus, sparse eyebrows and eyelashes, short neck, JH, low posterior hairline, brain MRI: abnormal myelinization, generalized MuHy and muscular hypotrophy, ataxia, dyssynergia, stereotypies, bruxism, strabismus
SEG2_33	F	Angelman syndrome (OMIM#105,830)	Global DD/ID, microcephaly, trigonocephaly, prominent forehead, prognathia, epicanthus, depressed nasal bridge, thin upper lip vermillion, high palate, short neck, shield chest, TPC, brain MRI: syringomyelia, Arnold-Chiari type I malformation, encephalopathy, epilepsy, generalized MuHy, ataxia, dyssynergia, incoordination, tongue thrusting, stereotypies, sleeping disrder, abnormal social behavior, strabismus, CALM, excessive sweating and salivation
SEG2_37	M	16q22.2q23.3 microdeletion [OMIM#614,541, (41)]	Somatic DD, global DD, large anterior fontanel, relative macrocepahly, triangular face, high, broad and prominent forehead, micrognathia, wide nasal bridge, low-set and crumpled ears, high palate, JH, TPC, brain MRI: mild ventriculomegaly, focal hyperintensities in frontal lobe, sleeping disrder, delayed eruption of teeth, 4 CALMs
SEG2_39	M	Proximal 16p11.2 microdeletion (OMIM#611,913)	Early obesity, global DD/ID, brachydactyly, stereotypies, abnormal emotion/affect behavior, poor attention, abnormal eating behavior, encopresis, impaired ability to form peer relationships, unilateral renal agenesis
SEG2_44	M	2q23.1 microduplication (49)	Pre- and postnatal overgrowth, global DD/ID, articulation disorder, relatively large facial skeleton, facial asymmetry, micro- and retrognathia, epicanthus, depressed nasal bridge, macrotia, abnormality of the pinna, linear earlobe crease, thick lower lip vermillion, hypoplastic philtrum, high palate, flat occiput, hypermobility of finger joints, stiffness of other joints, deep palmar and plantar creases, long toes, sandal gap, high anterior hairline, down-sloping shoulders, generalized MuHy, good memory, autistic features, umbilical hernia, increased bone age, scoliosis, gynecomastia, naevus flammeus
SEG2_49	M	14q11.2 microdeletion (20)	ID/DD, microcephaly, micrognathia, nasolacrimal duct stenosis, deep set eyes, depressed nasal bridge, bulbous nose, abnormality of the pinna, open mouth, unilateral STPC, brachydactyly, bilateral cutaneous syndactyly, bilateral cutaneous syndactyly of the 3rd -4th toes, scoliosis, flexion contractures of the fingers, axial hypotonia, limb hypertonia, incoordination, broad-based gait, cranial US: ventriculomegaly, hearing impairment, strabismus, delayed eruption of teeth
SEG2_50	F	8p23.1 microdeletion (ORPHA: 251,071)	Global DD, broad and prominent forehead, micro- and retrognathia, hypertelorism, depressed nasal bridge, low-set ears, high palate, pectus excavatum, kyphosis and lumbar hyperlordosis, congenital hip dislocation, broad 1st toe, JH, axial MuHy, normal brain MRI, reduced tendon reflexes, poor attention, strabismus, myopia, delayed eruption of teeth, capillary hemagioma, sacral dimple, atrial SD
SEG2_52	F	1p36 microdeletion [OMIM#607,872, (44)]	Feeding difficulties, global DD, large anterior fontanel, broad forehead, hypertelorism, low-set and simple ears, high palate, thin lip vermillions, short and hypoplastic philtrum, dorsalflexion pf the feet, bilateral TPC, overlapping toes, clinodactyly of the toes, low anterior hairline, hirsutism, brain MRI: ventriculomegaly, frontotemporal polymicrogyria, CCH, hypoplasia of brainstem and white matter, generalized MuHy, epilepsy, Fallot tetralogy
SEG2_53	F	Distal 16p11.2 microdeletion (OMIM#613,444)	Pre- and postnatal overgrowth, early obesity, global DD, genu varum, small hands and feet, generalized MuHy, polyphagia, abnormal social behavior, strabismus
SEG2_59	F	17p11.2 microdeletion (Smith-Magenis syndrome, OMIM#182,290)	Global DD, speech regression, triangular face, broad and high forehead, wide nasal bridge, open mouth, genu valgum, calcaneus valgus, broad 2nd fingers, clinodactyly of the 4th toes, brain MRI: arachnoid cysts hemosiderin deposits, broad-based gait, ataxia, epileptiform EEG, stereotypies, tongue thrusting, self-biting, aggression, abnormal eating behavior, assisted reproduction, healthy twin, strabismus, 1 CALM, naevus flammeus, excessive salivation, atrial SD, spleen cysts, GERD
SEG2_61	M	Léri-Weill dyschondrosteosis (OMIM#127,300), idiopathic short stature (OMIM#300,582)	Somatic DD, short limbs, global DD, wide and depressed nasal bridge, low-set and prominent ears, thin lip vermillions, short neck, flat occiput, small hands and feet, brachydactyly, brain MRI: ventricular asymmetry, generalized MuHy, tongue thrusting, impaired pain sensation, congenital stridor, scoliosis, tongue haemangioma, hepatomegaly, lymphangioma
SEG2_62	M	X-linked ichtyosis (OMIM#308,100) and Temple syndrome (UPD14mat, OMIM#616,222)	Somatic DD, global DD, relative macrocepahly, high and prominent forehead, low-set ears, high palate, clinodactyly of the 5th finger, TPC, brain MRI: partial empty sella, thin pituitay gland stalk, myopathic signs on EMG, decreased fetal movement, generalized MuHy, delayed bone age, micropenis, unilateral retractile testis, growth hormone deficiency, hypoglycaemia, generalized dry skin, discrete white scaling of the skin on extensor surfaces, ichthyotic skin on ears and scalp
SEG2_68	F	14q11.2 microdeletion (20)	Parents: first cousins, ID/DD, somatic DD, short stature, turricephaly, long face, thick eyebrows, abnormality of the pinna, hypodontia, long and slender fingers, absent speech, inability to walk, spasticity, muscular hypotrophia, epilepsy, brain MRI: cerebral atrophy, talipes equinovarus, hirsutism, hypothyroidism
SEG2_70	M	4q32.2q35.2 deletion (51)	Feeding difficulties, somatic DD, global DD, micro- and retrognathia, wide and depressed nasal bridge, low-set ears, preauricular skin tag, bilateral TPC, camptodactyly of 2nd -5th fingers, absent 5th distal phalngeal bones, overlapping toes, camptodactyly of 4th toes, brain MRI: choroid cysts, generalized MuHy, weak cry, cleft palate, glossoptosis, osteoarthritis (shoulder region), haemangioma, cardiomegaly, tracheal stenosis, hypospadias, double meatus urethrae, unilateral retractile testis, contralateral cryptorchidism, unilateral severe hydronephrosis, nephrolithiasis
SEG2_78	F	Xq22 microdeletion (50)	Prematurity, polyhidramnios, small fontanels, complicated perinatal adaptation, breathing difficulties in infancy, microcephaly, turricephaly, bitemporal narrowing, micrognathia, upslanted and short palpebral fissures, hypotelorism, depressed nasal bridge, abnormality of the pinna, high palate, webbed neck, low anterior and posterior hairlines, wide intermammillary distance, cutis laxa, stiff joints, ulnar deviation of the hands, overlapping fingers and toes, unilateral STPC, clinodactyly of the 5th fingers, camptodactyly of toes, sandal gap, flexion contractures of the fingers, dorsalflexed feet, hypoplasia of the nails, global DD, MuHy, poor visual behavior, reduced tendon reflexes, brain MRI: ventriculomegaly, cerebral atrophy, CCH, atrial SD, PS, absent right superior vena cava, persistent left superior vena cava, double aortic arch, GERD
SEG2_80	M	22q11.21q11.22 microdeletion (47)	SGA, prematurity, somatic DD, articulation disorder, microcephaly, long face, micrognathia, epicanthus, prominent nasal bridge, simple ears, high palate, crowded teeth, thin upper lip vermillion, brachydactyly, enuresis nocturna, umbilical hernia, mild conductive hearing impairment, submucous cleft palate, ankyloglossia, delayed bone age, dry skin, atrial SD, recurrent infections
SEG2_82	F	Phelan-McDermid syndrome (OMIM#606,232) and 19q13.43 microduplication (45,46)	Global DD, high palate, widely spaced teeth, conical teeth, broad thumbs and 1st toes, JH, normal brain MRI, generalized MuHy, ataxia, frequent falls, poor eye contact, hyperorality, MI, PI, recurrent infections
SEG2_85	F	16q12.2q21 microdeletion (40)	SGA, short stature, obesity, global DD/ID, narrow forehead, low-set ears, open mouth, macroglossia, high palate, brachydactyly, normal brain MRI, epilepsy, coarctation of the aorta, HCMP
SEG2_87	F	17q12 microduplication (OMIM#614,526)	Mild DD, high forehead, depressed nasal bridge, hypertelorism, low-set ears, atrial SD
SEG2_89	M	14q11.2 microdeletion (20)	Perinatal hypoxia, polyhidramnios, complicated perinatal adaptation, ID/DD, somatic DD, short forehead, deep-set eyes, synophris, short and hypoplastic philtrum, high palate, broad 1st finger, brachydactyly tapered fingers, unilateral STPC, broad hallux, overlapping toes, cutis laxa, Trendelenburg sign, tremor of the tongue, brain MRI: ventriculomegaly, tongue thrusting, umbilical hernia, refraction error, lumbar hyperlordosis, vertebral anomalies, hip dysplasia, hypertrichosis, bilateral pyelectasis, cryptorchidism, GERD

y: years; m: months; d: days; OMIM: Online Inheritance in Man;

ASD: autism spectrum disorder; CALM: café au lait macule; CCH: corpus callosum hypoplasia; DD: developmental delay; EMG: electromyogram; GERD: gastroesophageal reflux; HCMP: hypertrophic cardiomyopathy; ID: intellectual disability; JH: joint hypermobility; MI: mitral valve insufficiency; MRI: magnetic resonance imaging; MuHy: muscular hypotonia; PDA: patent ductus arteriosus; PI: pulmonary valve insufficiency; PS: pulmonary valve stenosis; SD: septal defect; SGA: small for gestational age; TPC: transverse palmar crease

Furthermore, we identified 24 variants of unknown significance (VUS) in 17 children (21.79% of the cohort); five individuals carried two, while one individual carried three VUS simultaneously (Additional file 4 lists detailed genetic and phenotypic information). Four children carried VUS in addition to disease-causing variants. The average size of the VUS was 585.9 Kb (median 228.2 Kb), 12 were losses and 12 were copy number gains (Additional file 2).

**Fig. 1:**
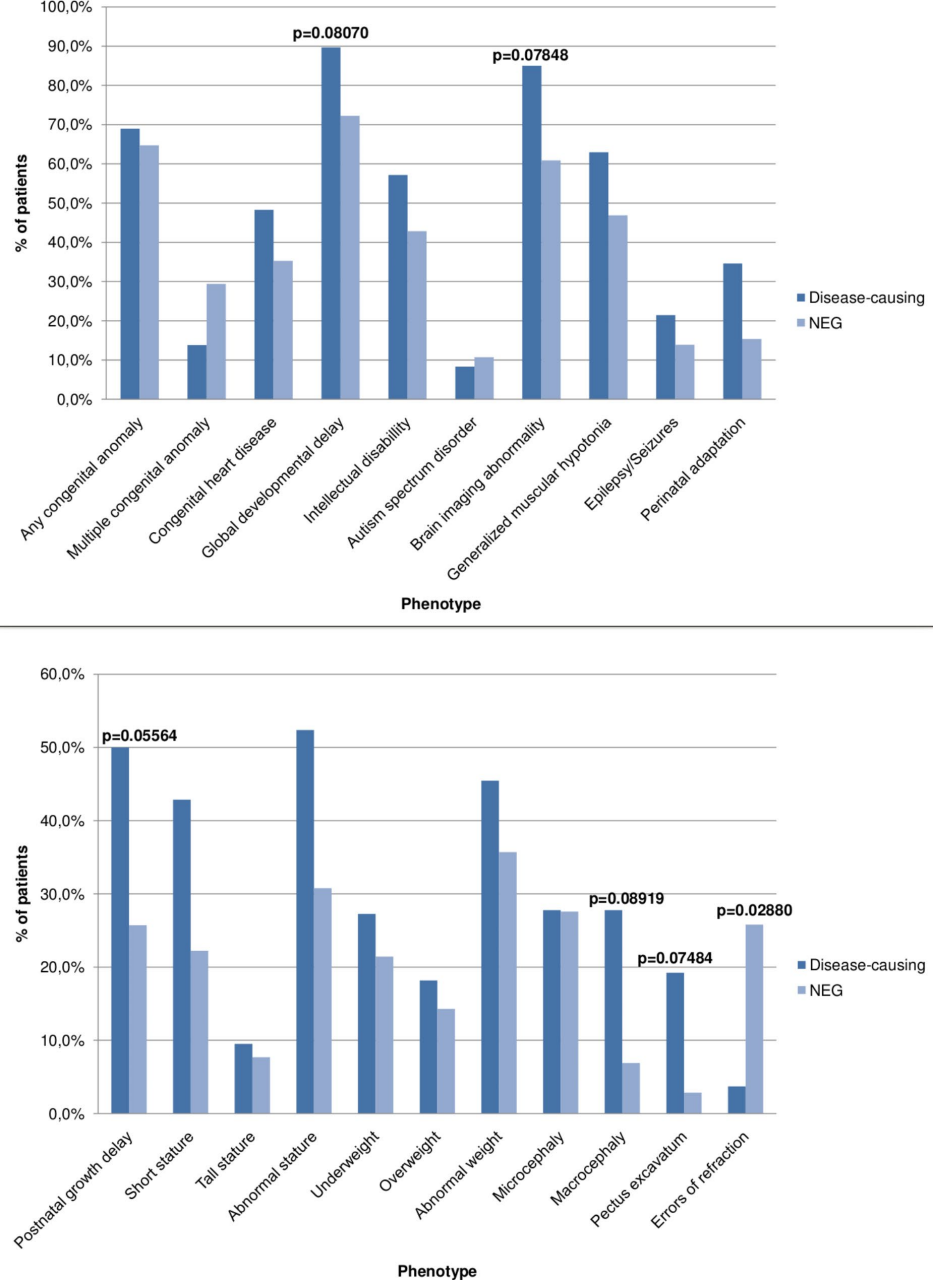
Phenotypic comparison of the patients with disease-causing variants and those with negative CMA results

We compared the main phenotypic features of the disease-causing CNV carrier patients and the negative CMA group (visualized on Fig. 1). Due to the patient selection criteria, NDDs were common in both groups, as were congenital anomalies of the internal organs. Postnatal growth delay was the only symptom to approach significance (p = 0.05564). Pectus excavatum (p = 0.07484), brain imaging abnormalities (p = 0.07848), global DD (0.08070), the sub-phenotype of speech and language delay (p = 0.08070) and macrocephaly (p = 0.08919) were more commonly, but none-significantly associated with disease-causing CNVs. Conversely, errors of refraction were more common in the negative group, the difference reached significance (p = 0.02880).

We were able to investigate inheritance in only 12.8% of patients; including three families where only the mother agreed to testing (neither mother carried the CNV of the respective child). Samples were available from both parents for seven patients. These seven children carried in total 9 CNVs, three proved to be *de novo* alterations, three were maternally, and three were paternally inherited. Six of the carrier parents were healthy, while the other four showed mild symptoms (history of learning difficulties and/or pediatric obesity, behavioral disorders, etc.; Additional file 5).

## Discussion

In our study, CMA enabled an etiologic diagnosis in 29 children in a cohort of 78 patients with NDDs and/or congenital anomalies. A relatively high proportion of VUS were identified as well. Our diagnostic yield (37.18%) is quite high compared to the estimated 15–20% yield of CMA methods, albeit these estimates are often based on studies where selection criteria were rather broad and CMA resolution was relatively low [2]. Our diagnostic yield is comparable to several studies with smaller patient groups and/or similarly strict selection criteria [5–15].

Our rigorous selection also impacted our statistical observations (keeping in mind the limitations of the small sample size as well). In contrast to our results, congenital heart defects and other major congenital anomalies are often observed in significantly higher frequencies in patients with definitive CMA results [3, 10, 16]. The association of postnatal growth delay, global DD, speech and language delay and brain imaging abnormalities is in line with literature data [3, 16, 17]. Macrocephaly and pectus excavatum are well-known suggestive features/minor anomalies, and are therefore not unexpected results. Only one child had an error of refraction in the disease-causing CNV group, in contrast to 7 patients in the negative group.

Chromosomes 16, 22, 14, 17 and X were most frequently affected by disease-causing imbalances, which aligns with literature data. Six of the nine chromosome 16 CNVs, two of the four imbalances of chromosome 22, two of three on chromosome 17 and one on chromosome X corresponded to recurrent microdeletion/microduplication regions, and thus are the consequence of non-allelic homologous recombination mediated by segmental duplications [18]. Amongst the 16p alterations, a noteworthy four were gains or losses of the proximal 16p11.2 region, which corresponds to 5.1% of the entire cohort, and 13.8% of children diagnosed. Recurrent rearrangements are known to present with highly variable phenotypes, and are incompletely penetrant [19] The presented patients’ phenotypes are in accordance with their respective recurrent rearrangements.

Surprisingly, microdeletions of a non-recurrent region were also slightly enriched in our study: 3/78 patients (3.8% of the studied cohort, 10.3% of diagnosed patients) carried approximately 500 Kb large deletions of 14q11.2. Microdeletions of chromosome region 14q11.2 are associated with DD/ID, ASD, other neuropsychiatric disorders, macrocephaly and characteristic facial features (hypertelorism, down-slanting palpebral fissures, broad nose, long philtrum, prominent Cupid’s bow and abnormalities of the auricular pinnae) [20, 21]. The authors of the original report [20] defined a minimal critical region (MCR) of 35 Kb encompassing two genes: *SUPT16H* (OMIM*605,012) and *CHD8* (OMIM*610,528). *CHD8* is currently thought to be one of the most common genetic drivers of ASD, and is often additionally associated with macrocephaly, overgrowth, facial minor anomalies and gastrointestinal problems [21–24]. *SUPT16H* was recently linked to a NDD consisting of DD/ID, ASD, seizures, precocious puberty, craniofacial minor anomalies, and corpus callosum abnormalities, but not macrocephaly [25]. Notably, neither of our three patients had increased head circumference; on the contrary, SEG2_49 had microcephaly (with generalized somatic underdevelopment). We speculate that this is due to other, as of yet unknown genetic factors counteracting the effect of the *CHD8* pathogenic alteration. Taken together with one other reported child with normal head circumference [26], our results highlight the fact that macrocephaly is not an obligatory symptom in SUPT16H-CHD8 microdeletions. Our patients enable further phenotypic expansion of the microdeletion: somatic DD/short stature, muscular hypertonia/spasticity, ventriculomegaly and hirsutism/hypertrichosis, deep-set eyes and STPC are all novel associated features noted in 2/3 patients in the current study (Table 2).

**Fig. 2:**
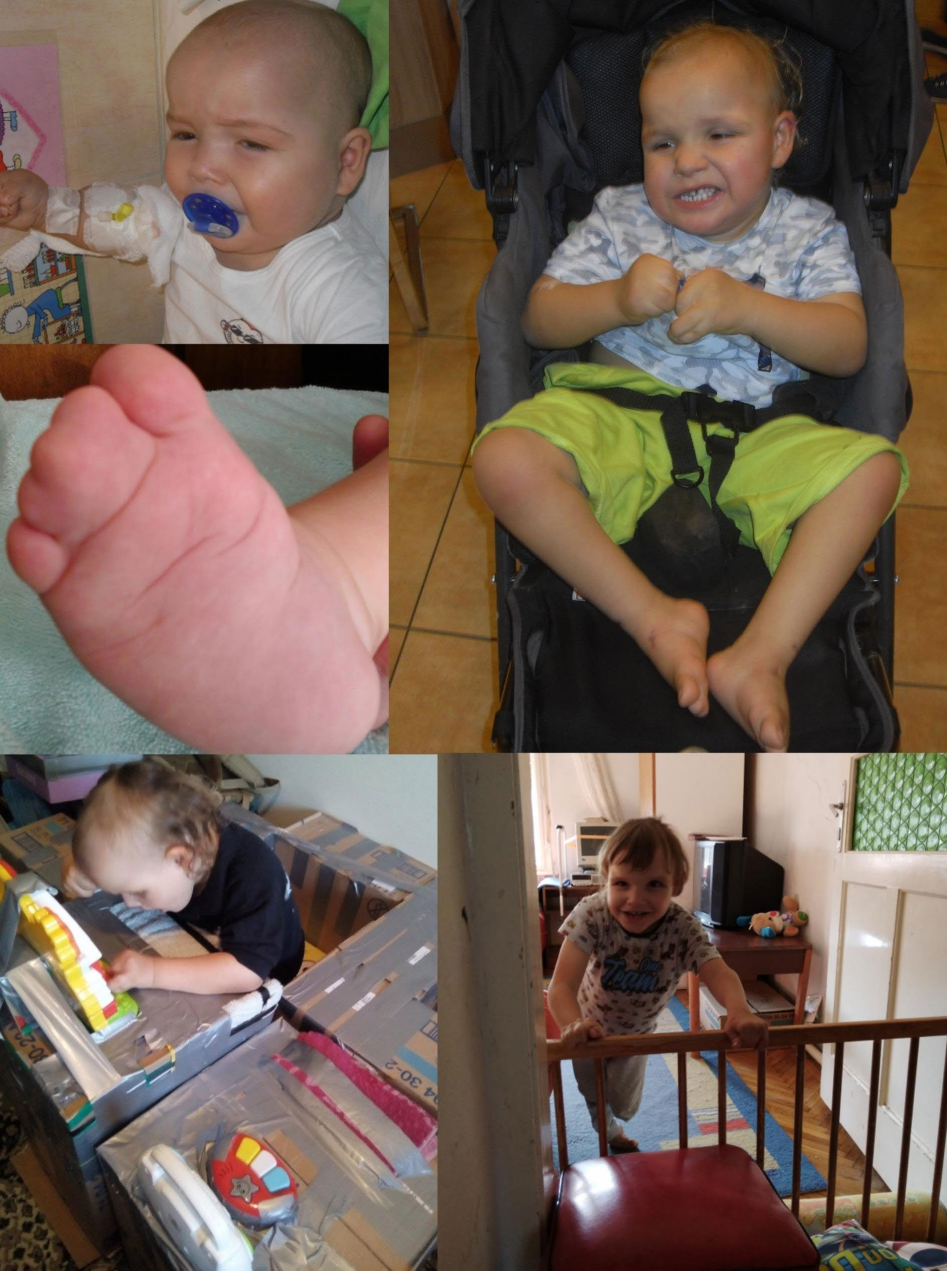
**Phenotype of patient SEG2_57** Left: close-up of one of the child’s feet (syndactyly and pes equinovarus). Right: age 3 years. At this age he was unable to sit without assistance. He learned to sit and hold himself up while leaning on something at age 5 years, and to pull himself to a standing position at age 7 years

To this day, classification of CNVs is often confounded by lack of sufficient evidence, and is thus dynamic as the accumulation of data, case reports, functional analyses, etc. can lead to reclassification of VUS. Improved genetic counseling and optimal patient care necessitates further studies to resolve the ambiguity of the not insignificant number of uncertain variants. Two of the VUS identified in the presented cohort merit further discussion. Patient SEG2_57 is a young boy with severe DD/ID, behavioral stereotypies and multiplex congenital anomalies (bilateral talipes equinovarus, bilateral complete syndactyly of the fingers and the toes, nail dystrophy, macrocephaly and postnatal overgrowth, craniofacial minor anomalies, spasticity, mild congenital heart disease, strabismus; see Fig. 2 and Additional file 4). The etiology of the child’s phenotype is complicated by two factors: there is a high likelihood of perinatal hypoxia; and four days after a syndactyly correcting operation he presented sudden loss of vision, multiple symptomatic focal epileptic attacks and diffuse hypoxic-ischaemic encephalopathy with cytotoxic cerebral oedema. This happened unexpectedly, and no cause has been identified to date (genetic variants causing malignant hyperthermia were ruled out from a blood sample, but were not tested from muscle biopsy). There were no further seizures from this point onward, and treatment (carbamazepine) was discontinued after one year. Pediatric neurologists retrospectively identified very mild cerebral atrophy when reevaluating the patient’s pre-surgery brain MRI. At the 6 month follow-up, MRI revealed severe cortical necrosis as sequelae to the encephalopathy, as well as diffuse mild cerebral atrophy. The boy has consistently slightly elevated lactate levels. His karyotype was determined to be 46,XY; pathogenic *GLI3* (OMIM*1,654,240) variants causing Greig cephalopolysyndactyly (OMIM#175,700) were ruled out at an early stage of genetic investigations; WES was performed later and identified no pathogenic variants or VUS that might explain the phenotype. Mitochondrial genome analysis performed on peripheral blood was negative. CMA analysis identified three VUS (Additional file 4), one of which encompasses an NDD-associated disease-causing gene. His 384 Kb large chromosome 4 duplication (arr[GRCh37]4q24(102058416_102443207)x3) contains *PPP3CA* (OMIM*114,105). The encoded calcineurin A protein (the catalytic subunit of calcineurin, which is responsible for calmodulin-calcineurin interactions) has an important role in synaptic vesicle recycling through regulation of response to calcium levels [27]; and is associated with epilepsy and other NDDs. Loss-of-function pathogenic variants cause autosomal dominant developmental and epileptic encephalopathy 91 (OMIM#617,711). This disorder is characterized by early-onset epilepsy (severity is variable and may be correlated with the specific variant), moderate/severe DD/ID, developmental regression, autistic behavior, generalized muscular hypotonia or spasticity, talipes equinovarus, feeding difficulties, cortical vision loss, cerebral atrophy and delayed myelinization [28–30]. Gain-of-function pathogenic variants lead to a syndrome characterized by arthrogryposis, cleft palate, craniosynostosis and ID (OMIM#618,265). Postnatal growth delay, plagio- or trigonocephaly, vesicoureteral reflux, gracile bones, brachydactyly, generalized seizures, behavioral stereotypies may also be present [31]. The other two VUS of this patient are a 504 Kb size 20p11.23 duplication (arr[GRCh37]20(19240620_19745197)x3), encompassing *SLC24A3* gene (OMIM*609,839), important for calcium homeostasis [32]; and an 81 Kb size Xq25 deletion (arr[GRCh37]X(124088718_124169834)x0), containing part of *TENM1* gene (OMIM*300,588), relevant in synaptic organization [33]. The phenotype of the presented SEG2_57 patient greatly overlaps with the disorders associated with *PPP3CA* (severe DD/ID, talipes equinovarus, cerebral atrophy, vision loss, and arthrogryposis, abnormal cranial morphology, behavioral stereotypies). The duplication of this gene plausibly contributed to the patient’s complex disorder, genotype-phenotype correlation is however confounded by the presence of multiple uncertain environmental factors. The syndromic origins of the child’s epilepsy and vision loss are questionable, but they might be attributable to decompensation of an existing genetic disorder. The complete syndactyly affecting all four extremities remains unexplained, which seems counter-intuitive at first glance, however, approximately 60% of syndactyly cases are sporadic, and hereditary cases are often isolated and multifactorial [34].

One further potentially relevant VUS is the 154 Kb large chromosome 20 duplication (arr[GRCh37]20p11.21(24554628_24708699)x3) encompassing *SYNDIG1* (OMIM*614,311) gene. This CNV was identified in a 5-year-old boy (patient SEG2_81, Additional file 4) who presented with moderate global developmental delay. His speech delay is particularly severe as he has no spoken words; receptive speech is also impaired, albeit to a lesser degree than expressive. He was initially diagnosed with ASD, but this diagnosis was later revised due to the complete failure of autism-specific developmental therapies and the overall longitudinal clinical picture. He is currently under the care of an expert neuropsychologist; extensive medical evaluations have unfortunately been repeatedly delayed. Preliminary neuropsychiatric opinion states that the patient has a complex NDD affecting the quality of many neurodevelopmental domains, including social interactions, behavior, and processing functions. A previous brain MRI (performed in 2017) was normal except for mild atrophy of the posterior third of the truncus corporis callosi. He has no other major or minor congenital anomalies. Patient SEG2_81’s 20p11.21 duplication was inherited from the father. The encompassed gene, *SYNDIG1*, is a brain-specific transmembrane protein proven to be a regulator of excitatory synapse development in rat brain [35]. Further study has shown that *SYNDIG1*-deficient excitatory synapses have impaired structure and function, thus suggesting an important role in normal synapse development [36]. No comparable duplications of *SYNDIG1* have been reported in the literature or online databases. In the presented case, paternal inheritance further complicates classification. Nevertheless, the currently suspected pathogenesis of patient SEG2_81’s phenotype implicates faulty synaptic development.

The main limitations of this study are the small sample size, the different CMA platforms utilized (not all patients were tested using SNP arrays, therefore LOHs suggestive of syndromes caused by UPDs could have been missed), and the lack of information regarding inheritance in the majority of patients.

## Conclusion

The diagnostic yield of CMA in our Hungarian cohort of pediatric patients with NDDs and/or congenital anomalies was 37.2%, promoting the continued usefulness of molecular cytogenetics. Our high diagnostic rate is partly due to rigorous patient selection criteria necessitated by availability and funding considerations. We identified several phenotypic features that increased the likelihood of finding a diagnosis in the patient group: postnatal growth delay, brain imaging abnormalities, global DD, macrocephaly and pectus excavatum. We highlight new clinical features associated with the rare, non-recurrent 14q11.2 microdeletion. Classification, and therefore genetic counseling of VUS is still confounded by the lack of scientific knowledge, highlighting the benefits of continued data sharing. We provide detailed phenotypic information corresponding to all VUS identified in our cohort. Amongst these, we identified two novel rare variants containing genes (*PPP3CA* and *SYNDIG1*, respectively) possibly relevant for the associated clinical phenotypes, bolstering said genes’ conceivable role in human disease. Further studies and case reports are necessary to elucidate the pathogenicity of the presented CNVs.

## Methods

Clinical data up to the point of genetic diagnosis/negative CMA result were retrospectively collected and organized. Karyotypes (at standard band resolution of 450–550) were determined by analysis of 20 Giemsa-stained metaphases each from standard 72-hour peripheral blood lymphocyte cultures. The platforms and analysis software used for CMA were NimbleGen Array (CGX 1.4 M) with NimbleGen MS 200 Microarray Scanner (30 patients; Roche NimbleGen Inc., Madison, WI, USA), Agilent qChip Post (60 K; 5 patients) and Agilent 180 K oligo-array (18 patients) with Agilent Genomic Workbench 7.0 (Agilent Technologies, Santa Clara, CA, USA), Affymetrix CytoScan Optima (300 K; 5 patients), Affymetrix CytoScan 750 K (17 patients), and Affymetrix CytoScan HD (3 patients) with Affymetrix Genechip Scanner or Chromosome Suite Ananlysis (ChAS) 4.0 (Thermo Fisher Scientific, Inc.; Waltham, MA, USA). CNV interpretation was based on guidelines published by the American College of Medical Genetics and Genomics (ACMG) [37, 38]. Relevant CNVs were validated using FISH or quantitative multiplex PCR of short fluorescent fragments (QMPSF) analysis. Parental studies were possible in 10 families: parents of three families and a mother of a fourth child underwent CMA, two sets of parents and two additional mothers were tested using QMPSF, and targeted FISH testing was performed for two parent pairs (Additional file 5). The main phenotypic features of the patients with disease-causing CNVs were compared to the patients with negative CMA results using the chi squared test. If any cell of the contingency table had an expected value less than five, the Fisher exact test was applied.

## Electronic supplementary material

Below is the link to the electronic supplementary material.

Supplementary Material 1

Supplementary Material 2

Supplementary Material 3

Supplementary Material 4

Supplementary Material 5

## Data Availability

Not applicable.

## Abbreviations

ACMG: American College of Medical Genetics and Genomics
ACMG: autism spectrum disorder
CHG: comparative genomic hybridization
CMA: chromosomal microarray
CNV: copy number variation
DSD: disorders of sexual development
FISH: fluorescent in situ hybridization
Kb: kilobase pairs
LOH: loss of heterozygosity
Mb: megabase pairs
NDD: neurodevelopmental disorder
OMIM: Online Inheritance in Man
PCR: polymerase chain reaction
QMPSF: quantitative multiplex PCR of short fluorescent fragments
SNP: single nucleotide polymorphism
VUS: variant of unknown significance.
WES: whole exome sequencing
