# Supplementary material for: Clinical evaluation of rare copy number variations identified by chromosomal microarray in a Hungarian neurodevelopmental disorder patient cohort
Source: Mol Cytogenet. 2022 Nov 1;15:47. doi: 10.1186/s13039-022-00623-z (PMC9623912; doi:10.1186/s13039-022-00623-z)
Supplement: Supplementary file 1 — Supplementary Material 1 [file 13039_2022_623_MOESM1_ESM.docx]

| **Patient** | **Karyotype** | **Copy Number Variation** | **Size (Kb)** | **Disorder/Classification** | **Phenotype** |
| --- | --- | --- | --- | --- | --- |
| SEG2_1 | 47,XY,+mar[60] /46,XY[40].ish idic r(22)(q11.1.q11.21)x4 | arr[GRCh37] 22q11.1q11.21 (17435645-18656678×3(17598642-17799783) × 4dn | 163.0; 201.1; 856.9 | Cat eye syndrome [OMIM#115470, (Haltrich et al. 2014)]  LP variants | Somatic DD, global DD, ID, mood disorder, enuresis nocturna, encopresis, facial asymmetry, micro- and retrognathia, wide nasal bridge, bulbous nose, low-set ears, MRI: chronic ischaemia and gliosis (left ACM region), mild PVL, maltrotation of the hippocampi, cortical dysplasia; complex febrile seizures, epilepsy, muscular hypotrophy, transposition of the great vessels, atrial SD, anal stenosis, liver cysts, GERD, chronic colitis, varicocele, neurogenic bladder, hyperinsulinemia, Duane anomaly, mild conductive hearing impairment, ekzema, recurrent infections, followed by palm/sole scaling |
| SEG2_4 | 46,XY (phenotypic female) | arr[GRCh37]16p11.2(29591326-30190029)x1 | 598.7 | 16p11.2 microdeletion (OMIM#611913)  P variant | IUGR, SGA, premature, feeding difficulties, macrocepahly, micro- and retrognathia, down-slanted palebral fissures, depressed nasal bridge, low-set ears, webbed neck, bell-shaped thorax, talipes equinovarus, genu varum, short limbs, brachydactyly, severe generalized MuHy, mild ventriculomegaly, neonatal apnoe, IU fractures, generalized edema, atrial SD, PDA, elevated total and free testosterone (female external genitalia, US showed normal uterus); death in infancy |

| SEG2_10 | 46,XY with complex chromosomal rearrangement affecting chromosome 1,2, 4, 5, 10, 17 | arr[GRCh37]2q14.3(123431180-124854926)x1 | 1423.7 | 2q14.3 microdeletion, complex chromosomal rearrangement (Lengyel et al. 2021b)  LP variant | Learning difficulties, mild motor DD, severe speech DD, feeding difficulties, early obesity, high forehead, down-slanted palebral fissures, low-set ears, thin upper lip vermillion, short neck, sandal gap, generalized MuHy, normal brain MRI, incoordination, stereotypies, poor attention, hyperactivity, polyphagia, amblyopia, tachycardia, GERD |
| --- | --- | --- | --- | --- | --- |
| SEG2_18 | 46,XX, SRY- (phenotypic male) | arr[GRCh37]17q24.3(69511806-69666935)x3 | 155.1 | 17q24.3 (*SOX9* regulatory region) microduplication (Pinti et al. 2019)  P variant | Hypospadias, bilateral ovotestis, Mullerian duct remnant instead of prostate |
| SEG2_19 | 46,XX, SRY- (phenotypic male) | arr[GRCh37]17q24.3(69577001-69618000)x3 | 41.0 | 17q24.3 (*SOX9* regulatory region) microduplication (Pinti et al. 2019)  P variant | Hypogonadotropic hypogonadism, gynecomastia, hypospadias, unilateral ovotestis with cysts, contralateral atrophic testis |
| SEG2_22 | 46,XX,t(X;10)(p21;p12) | arr[GRCh37]7q31.1(107692546-107849992)x3; arr[GRCh37]Xp22.11(22000870-22167026)x3 | 157.5;  166.2 | VUS variants | Apgar: 4/10/10, MA, lumbar hyperlordosis, rib exostosis, light sensitivity, motor DD, muscular dystrophy, MuHy, psuedohypertrophy, Trendelenburg sign, Gowers sign, panic disorder |
| SEG2_43 | 46,XX,der(X)t(X;19)(p11;p13) | - | - | - | Global DD/ID, umbilical hernia, triangular face, tall and broad forehead, micro- and retrognathia, starbismus, long eyelashes, wide nasal bridge, unilateral preauricular ear tag, high palate, hypoplastic philtrum, thin lip vermillions, joint hypermobility, brittle scalp hair, wide intermamillary distance, generalized MuHy, decreased muscle mass, brain MRI: hypoplasia of corpus callosum, ventriculomegaly, corpus pineale cyst, seizures |

| SEG2_58 | 47,XXX, SRY- | - | - | - | Gonadal dysgenesis, secondary amennorhea, bilateral ovarian tumor |
| --- | --- | --- | --- | --- | --- |
| SEG2_83 | 46,X,der(X)dup(X)(p10p22.1)[9]/ 45,X[6] | arr[GRCh37]7p22.3p21.3(42976-12448132)x3;  arr[GRCh37] Xp22.33(61091-4062749)x1;  arr[GRCh37]Xp22.23p11.22(4078736-53762693)x3 | 12405.2; 4001.7; 53762.7 | 7p22.3p21.3 microduplication (3); Xp22.33 microdeletion [OMIM#300830, (38)] and Xp22.23p11.2 duplication (40)  P variants | Webbed neck, short limbs, skeletal aplasia of the calvaria |
| SEG2_86 | 46,X,rec(X)del(X)(p22.3pter)ins(Y)  (q11.21q12)mat | arr[GRCh37]Xp22.33(61091-2676167)x1  arr[GRCh37]Yq11.2q12(14619835-59335913)x1 | 2615.1  44716.1 | Xp22.33 microdeletion (D’Ambrosio et al. 2019) and insertion of Yq11.21q12 long arm material  LP variants | Somatic DD, short stature, obesity, brachycephaly, broad face, short limbs, Madelung deformity, small hands and feet |
|  |  |  |  |  |  |

**Additional File 1. Genotypic and phenotypic data fo the excluded patients.**
Kb: kilobase; OMIM: Online Mendelian Inheritance in Man; P: pathogenic; LP: likely pathogenic; VUS: variant of uncertain significance;
ACM: a. cerebri media; DD: developmental delay; GERD: gastroesophageal reflux disease; IU: intrauterine; IUGR: intrauterine growth restriction; ID: intellectual disability; MuHy: muscular hypotonia; PDA: patent ductus arteriosus; PVL: periventricular leukomalacia; SD: septal defect; SGA: small for gestational age
