# Supplementary material for: Clinical evaluation of rare copy number variations identified by chromosomal microarray in a Hungarian neurodevelopmental disorder patient cohort
Source: Mol Cytogenet. 2022 Nov 1;15:47. doi: 10.1186/s13039-022-00623-z (PMC9623912; doi:10.1186/s13039-022-00623-z)
Supplement: Supplementary file 2 — Supplementary Material 2 [file 13039_2022_623_MOESM2_ESM.docx]

|  | **Disease-causing variants** | **VUS** |
| --- | --- | --- |
| Percent of deletions | 63.3% | 50.0% |
| Average size | 3481 Kb | 586 Kb |
| Median size | 1124 Kb | 228 Kb |
| < 300 Kb | 4 (13.3%) | 12 (37.5%) |
| 300 Kb – 1 Mb | 10 (33.3%) | 7 (29.2%) |
| 1-5 Mb | 10 (33.3%) | 5 (20.8%) |
| 5-10 Mb | 3 (10.0%) | 0 |
| >10 Mb | 3 (10.0%) | 0 |

**Additional file 2. Size comparison of disease-causing and clinically uncertain CNVs.**
