# Supplementary figures and images for: Clinical evaluation of rare copy number variations identified by chromosomal microarray in a Hungarian neurodevelopmental disorder patient cohort
Source: Mol Cytogenet. 2022 Nov 1;15:47. doi: 10.1186/s13039-022-00623-z (PMC9623912; doi:10.1186/s13039-022-00623-z)

## Slide 1
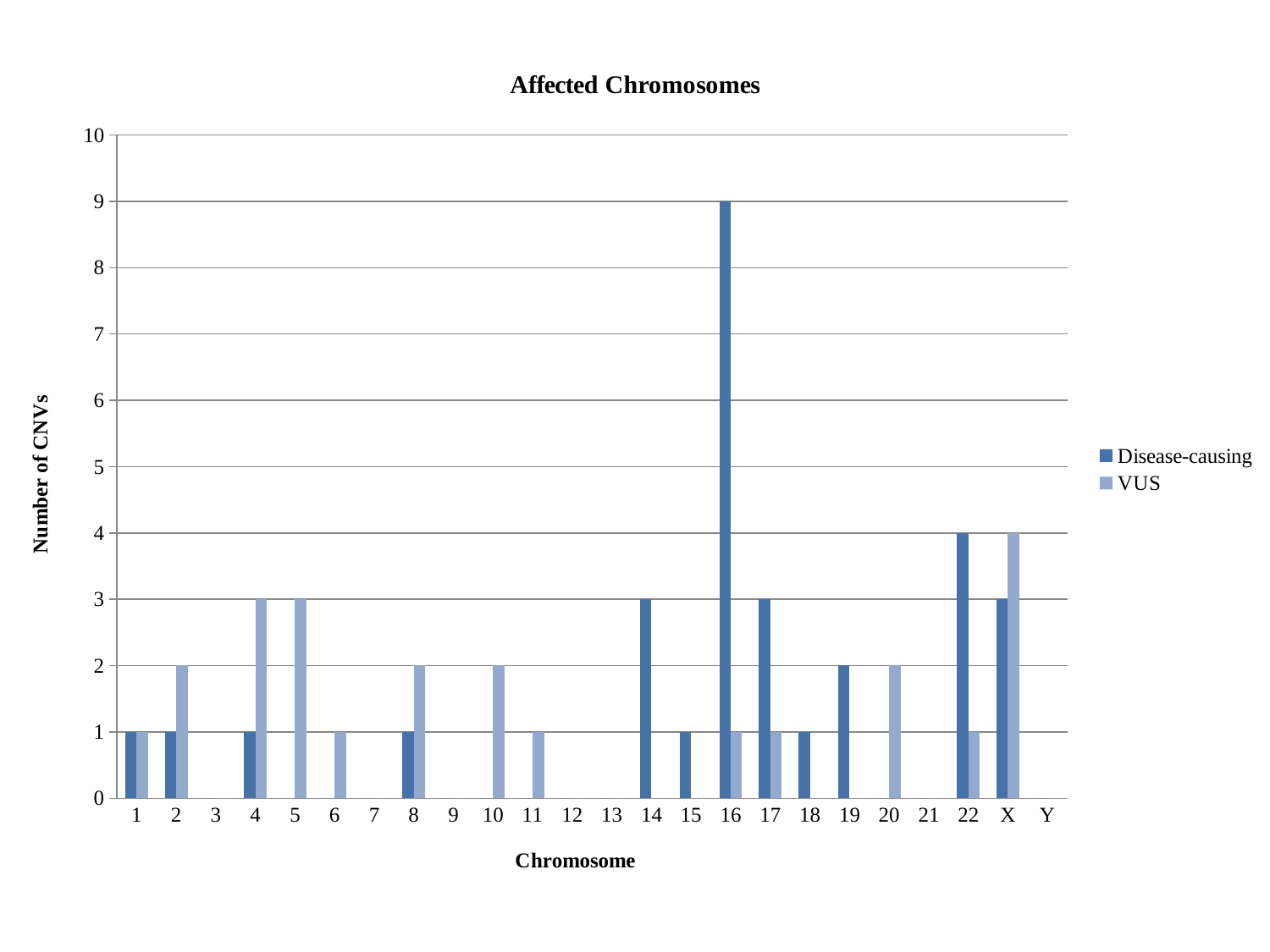

### Chart: Affected Chromosomes
| Category | Disease-causing | VUS |
|---|---|---|
| 1 | 1.0 | 1.0 |
| 2 | 1.0 | 2.0 |
| 3 | 0.0 | 0.0 |
| 4 | 1.0 | 3.0 |
| 5 | 0.0 | 3.0 |
| 6 | 0.0 | 1.0 |
| 7 | 0.0 | 0.0 |
| 8 | 1.0 | 2.0 |
| 9 | 0.0 | 0.0 |
| 10 | 0.0 | 2.0 |
| 11 | 0.0 | 1.0 |
| 12 | 0.0 | 0.0 |
| 13 | 0.0 | 0.0 |
| 14 | 3.0 | 0.0 |
| 15 | 1.0 | 0.0 |
| 16 | 9.0 | 1.0 |
| 17 | 3.0 | 1.0 |
| 18 | 1.0 | 0.0 |
| 19 | 2.0 | 0.0 |
| 20 | 0.0 | 2.0 |
| 21 | 0.0 | 0.0 |
| 22 | 4.0 | 1.0 |
| X | 3.0 | 4.0 |
| Y | 0.0 | 0.0 |

Supplement: Supplementary file 3 — Supplementary Material 3 [file 13039_2022_623_MOESM3_ESM.pptx]
