# Supplementary material for: Clinical evaluation of rare copy number variations identified by chromosomal microarray in a Hungarian neurodevelopmental disorder patient cohort
Source: Mol Cytogenet. 2022 Nov 1;15:47. doi: 10.1186/s13039-022-00623-z (PMC9623912; doi:10.1186/s13039-022-00623-z)
Supplement: Supplementary file 4 — Supplementary Material 4 [file 13039_2022_623_MOESM4_ESM.docx]

| **Patient** | **Sex** | **Copy Number Variation** | **Size (Kb)** | **Phenotype** |
| --- | --- | --- | --- | --- |
| SEG2_6 | M | arr[GRCh37]6q24.2(145089139-145133632)x1 | 44.4 | ID/DD, macrocephaly, MA, pectus excavatum, MuHy, intracranial cysts, stereotypies, inappropriate laughter |
| SEG2_9 | M | arr[GRCh37]4q34.1(172264978-173419929)x3 | 1155.0 | ID/DD, somatic DD, relative macrocephaly, MA, MuHy, corpus callosum atrophy, suspected PVL, seizures (normal EEG), aggression, ventricular SD |
| SEG2_23 | F | arr[GRCh37]5q13.3q14.1(75706732-78852164)x3 | 3145.4 | SGA, ID/DD, MA, strabismus, refraction error, JH, MuHy, ventriculomegaly, poor attention, ventricular SD |
|  |  | arr[GRCh37]5q23.2q31.1(122319833-126403246)x3 | 4083.4 |  |
| SEG2_24 | M | arr[GRCh37]2q13(110779468-111141038)x3 | 361.6 | Microcephaly, MA, speech DD, cryptorchidism, atrial SD, PS |
| SEG2_45 | M | arr[GRCh37]Xq28(153409765-153520551)x0 | 110.8 | ID/DD, weak and high-pitched cry, JH, polydactyly, MuHy, imbalance, social difficulties |
| SEG2_48 | M | arr[GRCh37]8p23.1(11145007-11291512)x1 | 146.5 | IUGR, neonatal breathing difficulties, ID/DD, somatic DD, microcephaly, MA, strabismus, nasal stenosis, axial hypotonia, limb hypertonia, CCH, articulation disorder, cryptorchidism, recurrent infections, atrial SD |
| SEG2_55 | M | arr[GRCh38]5q14.1(80253904-80965738)x3 | 711.8 | ID/DD, overgrowth, facial asymmetry, MA, MuHy, intracranial cysts, unilateral hydronephrosis and hydroureter |
|  |  | arr[GRCh38]10q11.22(46321708-46780831)x4 | 459.1 |  |
| SEG2_57 | M | arr[GRCh37]4q24(102058416-102443207)x3 | 384.8 | ID/DD, inability to walk, macrocephaly, MA, pes equinovarus, syndactyly, excessive sweating, cerebral atrophy, postoperative hypoxic-ischaemic encephalopathy and cytotoxic edema, cortical necrosis, ventriculomegaly, seizures, stereotypies, atrial SD, coarctation of the aorta, left ventricular hypertrophy, slightly elevated lactate |
|  |  | arr[GRCh37]20p11.23(19240620-19745197)x3 | 504.6 |  |
|  |  | arr[GRCh37]Xq25(124088718-124169834)x0 | 81.1 |  |
| SEG2_71 | F | arr[GRCh37]17q25.3(79989503-79992106)x1 | 2.6 | Oligohydramnios, IUGR, weak cry, global DD, somatic DD, MA, bilateral inguinal hernia, bilateral sensorineural hearing impairment, scoliosis, vertebral anomalies, hypoplastic hip bones, MuHy, incoordination, enuresis nocturna, atrial SD, PDA, pulmonary HT, gallbladder cysts, ectopic kidney |
| SEG2_72 | M | arr[GRCh37]11p13(36062989-36291232)x1 | 228.2 | ID/DD, facial asymmetry, MA, excessive salivation, muscular hypotrophia, stereotypies |
| SEG2_75 | F | arr[GRCh38]2p25.1(10617430-10795996)x3 | 178.6 | ID/DD, MA, sensorineural hearing impairment, scoliosis, JH, genu valgum, calcaneus valgus, pes planus, MuHy, ventriculomegaly, incoordination, autistic behavior, poor attention |
|  |  | arr[GRCh38]4q13.3(70295481-70415836)x1 | 120.4 |  |
| SEG2_77 | M | arr[GRCh37]16p11.2(31980001-33825000)x1 | 1845.0 | Motor DD, MA, scoliosis, JH, articulation disorder, ASD, ADHD, temper tantrums, tracheal and anal stenosis |
|  |  | arr[GRCh37]Xq28(153409765-153520551)x0 | 110.8 |  |
| SEG2_81 | M | arr[GRCh37]20p11.22(24554628-24708699)x3 | 154.1 | ID/DD, autistic behavior, complex NDD |

**Additional File 4. Variants of unknown significance and patient phenotypes.**
ADHD: attention deficit hyperactivity disorder; ASD: autism spectrum disorder; CCH: corpus callosum hypoplasia; GERD: gastroesophageal reflux disease; ID/DD: intellectual disability/developmental delay; IUGR: intrauterine growth restriction; HT: hypertension; JH: joint hypermobilty; Kb: kilobase; MA: minor anomalies; MuHy: muscular hypotonia; NDD: neurodevelopmental disorder; PDA: patent ductus arteriosus; PS: pulmonary stenosis; PVL: periventricular leukomalacia; SD: septal defect; SGA: small for gestational age
