# Supplementary material for: Clinical evaluation of rare copy number variations identified by chromosomal microarray in a Hungarian neurodevelopmental disorder patient cohort
Source: Mol Cytogenet. 2022 Nov 1;15:47. doi: 10.1186/s13039-022-00623-z (PMC9623912; doi:10.1186/s13039-022-00623-z)
Supplement: Supplementary file 5 — Supplementary Material 5 [file 13039_2022_623_MOESM5_ESM.docx]

| **Patient** | **Copy Number Variation** | **Size (Kb)** | **Classification** | **Inheritance** | **Method** | **Parental phenotype** |
| --- | --- | --- | --- | --- | --- | --- |
| SEG2_5 | ^#^16p13.3(3263725-4309863)x1 | 1046,1 | P | de novo | FISH  Probe: Rubinstein Taybi^a^ | Healthy |
| SEG2_15 | 19p13.3(753219-1477508)x3  5p15.33p15.32(4260205-5088435)x3 | 724,3  828,2 | LP  VUS (LB) | de novo  pat | CMA | Healthy |
| SEG2_26 | 16p11.2(29624765-30199351)x3 | 574,6 | P | mat | CMA | Mat: Learning, social and behavioral difficulties |
| SEG2_27 | 16p11.2(29624765-30199351)x3 | 574,6 | P |  |  |  |
| SEG2_53 | 2q37(242855645-243030854)x1  16p11.2(28824802-29040571)x1 | 175,2  215,8 | LB  P | pat  mat | FISH  Probes: ATXN2L/SH2B1^b^; subtel 2q | Pat: learning difficulties, early obesity Mat: early obesity |
| SEG2_77 | 16p11.2(31980001-33825000)x1 | 1845,0 | VUS | de novo | QMPSF | Healthy |
| SEG2_81 | 20p11.21(24554628-24708699)x3 | 154,1 | VUS | pat | QMPSF | Pat: neuropsychiatric symptoms |
|  | | | | | | |
| SEG2_17 | 16p11.2(29620689-30190568)x3 | 569,9 | P | unk.* | QMPSF | Healthy |
| SEG2_39 | 16p11.2(29656684-30190568)x1 | 533,9 | P | unk.* | QMPSF | Healthy |
| SEG2_57 | 4q24(102058416-102443207)x3 | 384,791 | VUS | unk.* | CMA | Healthy |
|  | 20p11.23(19240620-19745197)x3 | 504,6 | VUS | unk.* |  |  |
|  | Xq25(124088718-124169834)x0 | 81,1 | VUS | unk.* |  |  |

**Additional file 5. Inheritance of the CNVs in the presented patient group**Kb: kilobase; P: pathogenic; LP: likely pathogenic; VUS: variant of unknown significance; LB: likely benign; mat: maternal; pat: paternal; QMPSF: quantitative multiplex PCR of short fluorescent fragments; FISH: fluorescent *in situ* hybridisation; CMA: chromosomal microarray; ^#^: GRCh38; all other genomic coordinates are according to GRCh37; unk.*: unknown; maternal inheritance was ruled out; ^a^: Cytocell Technologies, Ltd., Cambridge, UK; ^b^: Agilent Technologies, Santa Clara, CA, USA
